# A276 CANNABINOID RECEPTOR 1 IS LOCALIZED IN ENTERIC NEURONS AND EPITHELIUM IN FETAL AND EARLY POSTNATAL MOUSE INTESTINE

**DOI:** 10.1093/jcag/gwac036.276

**Published:** 2023-03-07

**Authors:** M Sunil, M Adnan, S Farcas, A Pugazhendhi, E Ratcliffe

**Affiliations:** 1 Department of Pediatrics; 2 Farncombe Family Digestive Health Research Institute; 3 Division of Gastroenterology and Nutrition, Department of Pediatrics; 4 Michael G. DeGroote Centre for Medicinal Cannabis Research, McMaster University , Hamilton, ON, Canada

## Abstract

**Background:**

The endocannabinoid system (ECS) is necessary for gastrointestinal (GI) homeostasis and serves as a target for exogenous medicinal and recreational cannabinoid compounds. While components of the ECS, including receptors, ligands, and biosynthetic/degradative enzymes, have been increasingly characterized in the adult GI tract of both humans and animal models, similar advances in understanding the role of the ECS in GI developmental programming have not yet been made.

**Purpose:**

We tested the hypothesis that the ECS plays a role in the developmental programming of the GI tract.

**Method:**

The developmental timeline of ECS component expression was investigated in the GI tract from fetal and early postnatal mice. Samples from timed-pregnant outbred mice were collected from embryonic days 14 (E14), E16, E18 and postnatal days 1 (P1), P7 and P28, representing a timeline of early colonization of the GI tract by enteric neurons (E14) to 1 week after weaning (P28). Localization of cannabinoid receptor 1 (CB1) expression was examined in fetal and postnatal small intestine by immunohistochemistry. Fetal intestine was processed for RNA extraction and relative expression of ECS components determined by RT-qPCR.

**Result(s):**

CB1 expression at E14 was observed in the outer wall of the gut and in the developing mucosa. By E18, expression of CB1 became more localized to the enteric plexuses and to mucosal epithelial cells. In postnatal ileum, at least >50% of myenteric ganglia were found to express CB1 from P1 to P28. CB1 expression continued to be present in the mucosal epithelium of ileum from P1 to P28, with a subset of CB1 expression in enterochromaffin cells, as identified by 5-HT immunolabeling. Significant decreases in expression of mRNA encoding CB1 (p<0.0001) and the ECS biosynthetic enzymes NAPE-PLD (p<0.05) and DAGLa (p<0.05) were found between E14 and E18.

**Conclusion(s):**

Components of the ECS are present in the fetal and early postnatal GI tract. Further research is needed to better characterize the roles and mechanisms of action of the ECS in GI development. The potential impact of exogenous cannabis exposure in early life, either by *in utero* exposure or via the use of medicinal cannabinoids in childhood, highlights the importance of identifying the contributions of the ECS to normal development.

**Disclosure of Interest:**

None Declared

